# Supplementary material for: Atypical flagella assembly and haploid genome coiling during male gamete formation in Plasmodium
Source: Nat Commun. 2023 Dec 13;14:8263. doi: 10.1038/s41467-023-43877-w (PMC10719364; doi:10.1038/s41467-023-43877-w)
Supplement: Supplementary file 1 — Supplementary Information [file 41467_2023_43877_MOESM1_ESM.pdf]

Microgametocyte organelles:

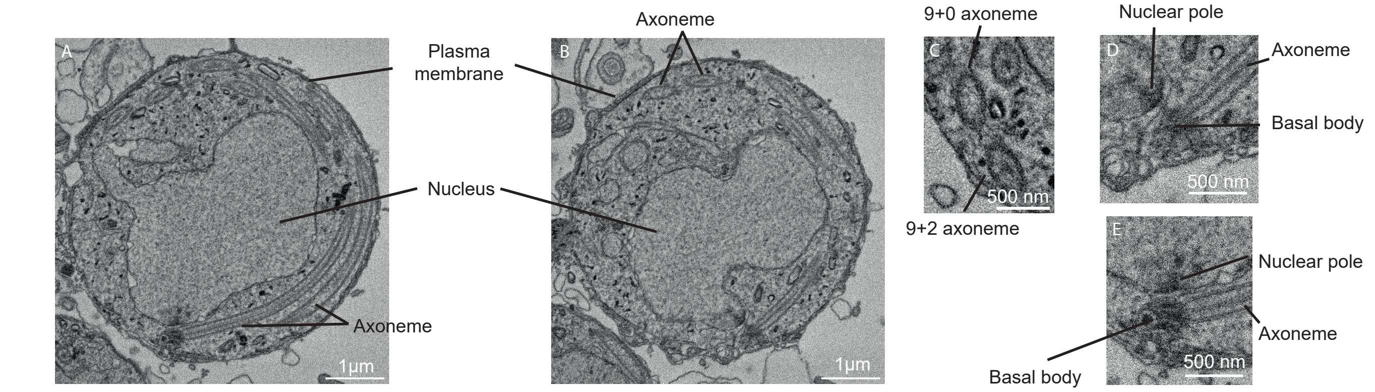


Microgamete organelles:

Proximal end:

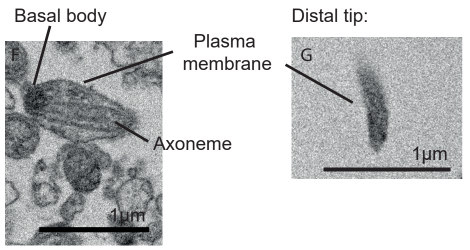


Serial slices through an SBF-SEM dataset to show a portion of the microgamete axoneme with a coiling nucleus:

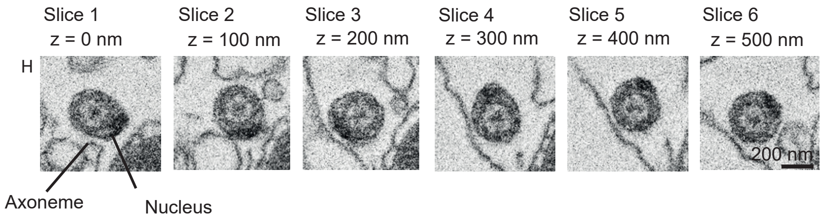


Each slice represents z of 100nm

**Supplementary Figure 1. SBF-SEM key to image segmentation**. (A-E) SBF-SEM data slices of microgametocyte organelles. (A-B) Whole microgametocyte cells showing example images of the plasma membrane, nucleus and axoneme. (C) Ultrastructural features used to define the outer doublets and central pair present (9+2) and absent (9+0) in the axoneme of microgametocytes. (D-E) Ultrastructural features used to define the electron dense nuclear pole positioned inside the nucleus and the electron dense basal body positioned on the cytoplasmic face of the nucleus and axoneme in microgametocytes. (F-G) SBF-SEM data slices of microgametes showing example images of the proximal end containing an electron dense basal body, plasma membrane and axoneme, and the distal tip. (H) Serial SBF-SEM data slices to show the axoneme coiling around the nucleus in a microgamete. Each slice represents a z slice of 100 nm.

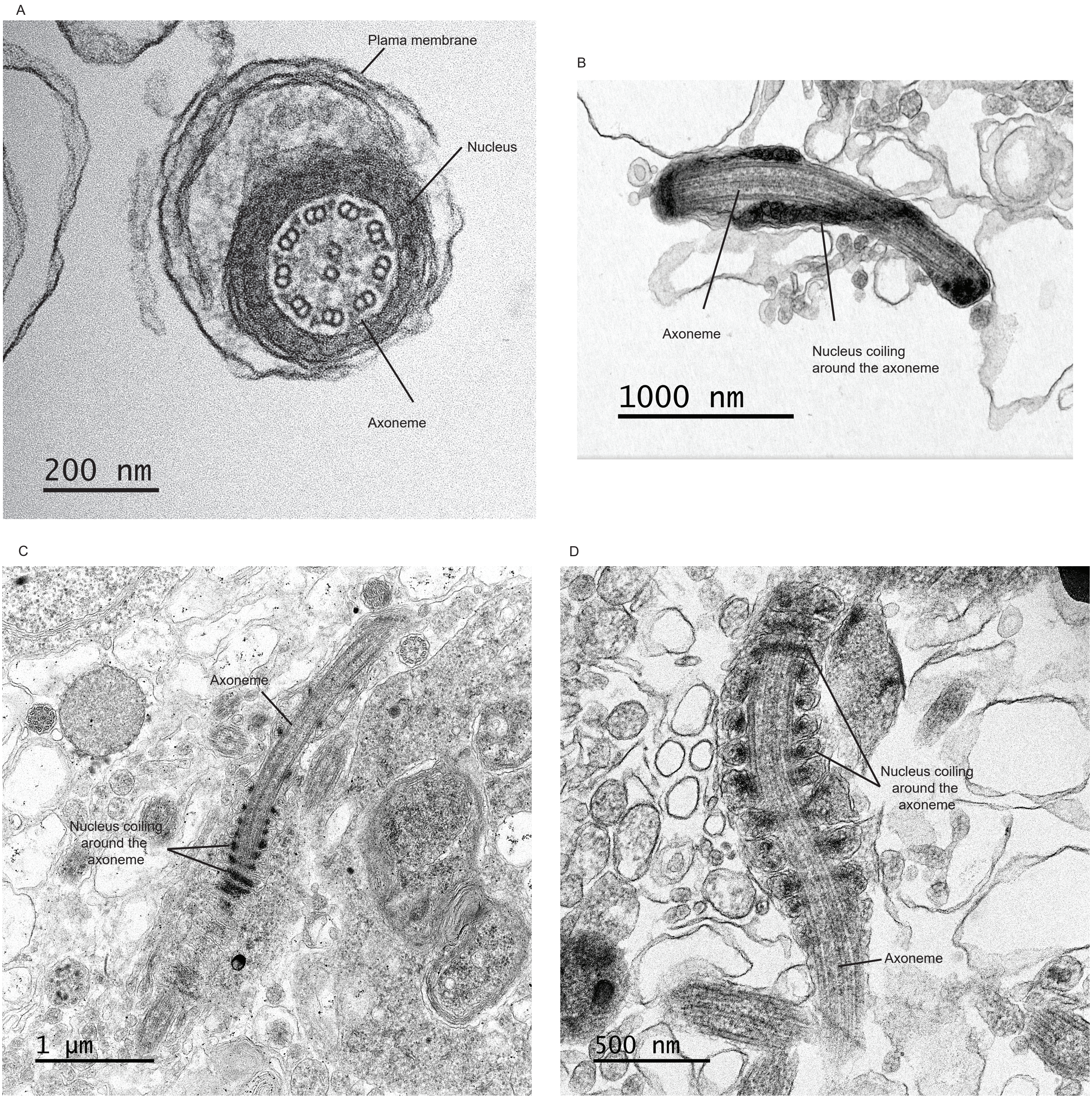
A
Plama membrane
Nucleus
Axoneme
200 nm
B
Axoneme
Nucleus coiling around the axoneme
1000 nm
C
Axoneme
Nucleus coiling around the axoneme
1 µm
D
Nucleus coiling around the axoneme
Axoneme
500 nm

**Supplementary Figure 2. Coiling of the nucleus around the axoneme in microgametes** (A-D) Random thin section transmission electron micrographs of microgametes highlighting the coiling of the nucleus around the axoneme.

Supplementary Table 1: Primers used in this study

| Primers used for SAS4 tag construct | | |
|---|---|---|
| Name | Sequence (5’ to 3’) | Notes |
| T2011 | CCCCGGTACCGAAAGTATGAGTTCAAGCAGTAATATG | KpnI site underlined |
| T2012 | CCCCGGGCCCATTCAACCTTTTTATAACACCTACAAC | ApaI site underlined |
| IntT201 | GTAGTGACACTAGTGTCTATTTAC | |
| ol492 (2) | ACGCTGAACTTGTGGCCG | |

| Primes ued for NDC80 tag construct | | |
|---|---|---|
| Name | Sequence (5’ to 3’) | Notes |
| T2591 | CCCCGGTACCGCGAATTCTAATAATAGGTTTAATC | KpnI site underlined |
| T2592 | CCCCGGGCCCTTCAGTTACACTTTGATGTAAATTTTTATATAATTC | ApaI site underlined |
| IntT259 | GATCTGGAAGAATCAATCAGAAAGAC | |
| mCherry | TTCAGCTTGGCGGTCTGGGT | |

| Primers used for Kinesin-8B tag construct | | |
|---|---|---|
| Name | Sequence (5’ to 3’) | Notes |
| T1991 | CCCCGGTACCCACACCATTTTATAATATCTGAATCTA | KpnI site underlined |
| T1992 | CCCCGGGCCCAATTTTATTTTTTATAATGTTAAAAAGATTTGAGAT | ApaI site underlined |
| Int199 | GTTGTTACATCTAATTCTGTTGTAG | |
| ol492 | ACGCTGAACTTGTGGCCG | |

**Supplementary Table 1. Oligonucleotides used to generate tagged *Plasmodium berghei* cell lines.**

**Supplementary Movie 1.** Serial block face scanning electron microscopy datasets used in this work. The section thickness (Z resolution) is 100 nm, and each dataset consist of 197 images (pixel dimension and X-Y resolution).

**Supplementary Movie 2.** SBF-SEM data and segmentation of a single microgametocyte post-activation showing 8 axonemes coiling around the nucleus in two orientations. Organelles are modelled on the following colours: plasma membrane (white), nucleus (blue), basal body (red), axonemes (yellow/blue – two colours to highlight different directions of coiling).

**Supplementary Movie 3.** SBF-SEM imaging and 3D model of a microgamete cell undergoing exflagellation. Structures are modelled on the following colours: plasma membrane (white), nucleus (cyan), basal body (red), flagella (pink).

**Supplementary Movie 4.** Serial tomogram of a microgamete highlighting the coiling of the nucleus around the axoneme.

**Supplementary Movie 5.** Serial tomogram of a microgametocyte showing a nuclear projection surrounding the axoneme, with the 3D reconstruction of the nucleoplasm (cyan), which forms a coil within the nuclear projection.
