# Supplementary material for: Atypical flagella assembly and haploid genome coiling during male gamete formation in Plasmodium
Source: Nat Commun. 2023 Dec 13;14:8263. doi: 10.1038/s41467-023-43877-w (PMC10719364; doi:10.1038/s41467-023-43877-w)
Supplement: Supplementary file 3 — Description of Additional Supplementary Files [file 41467_2023_43877_MOESM3_ESM.pdf]

Description of Additional Supplementary Files

File Name: Supplementary Movie 1
Description: Serial block face scanning electron microscopy datasets used in this work. The section thickness (Z resolution) is 100 nm, and each dataset consist of 197 images (pixel dimension and X-Y resolution).

File Name: Supplementary Movie 2
Description: SBF-SEM data and segmentation of a single microgametocyte post-activation showing 8 axonemes coiling around the nucleus in two orientations. Organelles are modelled on the following colours: plasma membrane (white), nucleus (blue), basal body (red), axonemes (yellow/blue – two colours to highlight different directions of coiling).

File Name: Supplementary Movie 3
Description: SBF-SEM imaging and 3D model of a microgamete cell undergoing exflagellation. Structures are modelled on the following colours: plasma membrane (white), nucleus (cyan), basal body (red), flagella (pink).

File Name: Supplementary Movie 4
Description: Serial tomogram of a microgamete highlighting the coiling of the nucleus around the axoneme.

File Name: Supplementary Movie 5
Description: Serial tomogram of a microgametocyte showing a nuclear projection surrounding the axoneme, with the 3D reconstruction of the nucleoplasm (cyan), which forms a coil within the nuclear projection.
