# Supplementary material for: Atypical flagella assembly and haploid genome coiling during male gamete formation in Plasmodium
Source: Nat Commun. 2023 Dec 13;14:8263. doi: 10.1038/s41467-023-43877-w (PMC10719364; doi:10.1038/s41467-023-43877-w)
Supplement: Supplementary file 9 — Reporting Summary [file 41467_2023_43877_MOESM9_ESM.pdf]

nature portfolio

Corresponding author(s): Sue Vaughan and Rita Tewari

Last updated by author(s): Oct 10, 2023

Reporting Summary

Nature Portfolio wishes to improve the reproducibility of the work that we publish. This form provides structure for consistency and transparency in reporting. For further information on Nature Portfolio policies, see our Editorial Policies and the Editorial Policy Checklist.

Statistics

For all statistical analyses, confirm that the following items are present in the figure legend, table legend, main text, or Methods section.

n/a	Confirmed	
☐	☒	The exact sample size (*n*) for each experimental group/condition, given as a discrete number and unit of measurement
☒	☐	A statement on whether measurements were taken from distinct samples or whether the same sample was measured repeatedly
☐	☒	The statistical test(s) used AND whether they are one- or two-sided<br>*Only common tests should be described solely by name; describe more complex techniques in the Methods section.*
☒	☐	A description of all covariates tested
☒	☐	A description of any assumptions or corrections, such as tests of normality and adjustment for multiple comparisons
☐	☒	A full description of the statistical parameters including central tendency (e.g. means) or other basic estimates (e.g. regression coefficient) AND variation (e.g. standard deviation) or associated estimates of uncertainty (e.g. confidence intervals)
☐	☒	For null hypothesis testing, the test statistic (e.g. *F*, *t*, *r*) with confidence intervals, effect sizes, degrees of freedom and *P* value noted<br>*Give P values as exact values whenever suitable.*
☒	☐	For Bayesian analysis, information on the choice of priors and Markov chain Monte Carlo settings
☒	☐	For hierarchical and complex designs, identification of the appropriate level for tests and full reporting of outcomes
☒	☐	Estimates of effect sizes (e.g. Cohen's *d*, Pearson's *r*), indicating how they were calculated

Our web collection on statistics for biologists contains articles on many of the points above.

Software and code

Policy information about availability of computer code

Data collection	All the widefield microscopy images were captured using a 63x oil immersion objective on a Zeiss Axio Imager M2 microscope fitted with an AxioCam ICc1 digital camera.<br>Serial block face scanning electron microscope datasets were produced using a Merlin VP compact high resolution scanning electron microscope (Zeiss) equipped with a 3View stage (Gatan-Ametek), and an OnPoint back-scattered electron detector (Gatan-Ametek).<br>Serial-section cellular electron tomography and transmission electron microscopy was performed on a JOEL JEM-1400 Flash transmission electron microscope.
Data analysis	All the widefield microscopy images were analysed with the AxioVision 4.8.2 software.<br>SBF-SEM datasets and tomography data were processed and analysed using the IMOD software package version 4.11.<br>Transmission electron micrographs were analysed using Fiji-ImageJ software.

For manuscripts utilizing custom algorithms or software that are central to the research but not yet described in published literature, software must be made available to editors and reviewers. We strongly encourage code deposition in a community repository (e.g. GitHub). See the Nature Portfolio guidelines for submitting code & software for further information.

Data

Policy information about availability of data

All manuscripts must include a data availability statement. This statement should provide the following information, where applicable:

- Accession codes, unique identifiers, or web links for publicly available datasets
- A description of any restrictions on data availability
- For clinical datasets or third party data, please ensure that the statement adheres to our policy

The SBF-SEM datasets generated and analysed for this study are available in the EMPIAR repository 26, EMPIAR ID: EMPIAR -11710. The supplementary movies generated for this study are available at Zenodo https://zenodo.org/record/8416867.

Research involving human participants, their data, or biological material

Policy information about studies with human participants or human data. See also policy information about sex, gender (identity/presentation), and sexual orientation and race, ethnicity and racism.

Reporting on sex and gender	N/A
Reporting on race, ethnicity, or other socially relevant groupings	N/A
Population characteristics	N/A
Recruitment	N/A
Ethics oversight	N/A

Note that full information on the approval of the study protocol must also be provided in the manuscript.

Field-specific reporting

Please select the one below that is the best fit for your research. If you are not sure, read the appropriate sections before making your selection.

☒ Life sciences ☐ Behavioural & social sciences ☐ Ecological, evolutionary & environmental sciences

For a reference copy of the document with all sections, see nature.com/documents/nr-reporting-summary-flat.pdf

Life sciences study design

All studies must disclose on these points even when the disclosure is negative.

Sample size	The sample sizes are described below. The mice numbers were calculated with the 3Rs (Replacement, refinement and reduction) in mind to produce meaningful, statistically valid and reproducible results as given below, and are based on our extensive experience in this area.<br>3 mice were used to generate each dual lines (two dual lines). Each respective gene GFP and mCherry lines were crossed within the mosquito and dual lines were obtained by mosquito bite back experiment. (SAS4/NDC80; SAS4/Kinesin8B)<br>2 mice/sample were used to capture for the live cell imaging.<br>5 mice / sample were used for SBF-SEM analysis to prepare purified gametocyte samples at two time points used in the study.
Data exclusions	Those data are excluded where we needed to optimize the experiments.
Replication	The experiments were repeated three times. All of them were successful in terms of imaging.
Randomization	The data analysis was done randomly by different researcher for collecting images.
Blinding	Parasites were genotyped for the gene targeting but this information was not written on the cage cards or accessible to the staff phenotyping the parasite so the parasites are assessed blind. For the imaging, the parasites were imaged without knowing the phenotype and time points. Most of the experiments were performed and analyzed by different groups blindly. For example, widefield microscopy was done at University of Nottingham and SBF SEM was done at Oxford Brookes University.

Reporting for specific materials, systems and methods

We require information from authors about some types of materials, experimental systems and methods used in many studies. Here, indicate whether each material, system or method listed is relevant to your study. If you are not sure if a list item applies to your research, read the appropriate section before selecting a response.

Materials & experimental systems

n/a	Involved in the study	
☒	☐	Antibodies
☒	☐	Eukaryotic cell lines
☒	☐	Palaeontology and archaeology
☐	☒	Animals and other organisms
☒	☐	Clinical data
☒	☐	Dual use research of concern
☒	☐	Plants

Methods

n/a	Involved in the study	
☒	☐	ChIP-seq
☒	☐	Flow cytometry
☒	☐	MRI-based neuroimaging

Animals and other research organisms

Policy information about studies involving animals; ARRIVE guidelines recommended for reporting animal research, and Sex and Gender in Research

Laboratory animals	Six- to eight-week-old female CD1 outbred mice from Charles River laboratories were used for all experiments. The conditions of mice kept are a 12hour light and 12hour dark (7 till 7) light cycle, the room temperature is kept between 20-24 degrees celcius and the humidity is kept between 40-60%.
Wild animals	NA
Reporting on sex	In this study it was not important to choose either female or male mice because we used them as a media/vehicle to grow Plasmodium and did not study the effect of infection on mice. The important part of the experiment was to grow the parasite in mice with sustainable parasitaemia that could be easily handled and managed. Plasmodium grew exactly the same way in both sexes, which we have tested previously. Here, we used female mice without any specific reason but found them less aggressive and easy to handle.
Field-collected samples	No field collected samples were used in the study.
Ethics oversight	The animal work passed an ethical review process and was approved by the United Kingdom Home Office. Work was carried out under UK Home Office Project Licenses (30/3248 and PDD2D5182) in accordance with the UK 'Animals (Scientific Procedures) Act 1986'.

Note that full information on the approval of the study protocol must also be provided in the manuscript.
